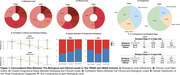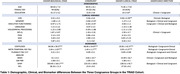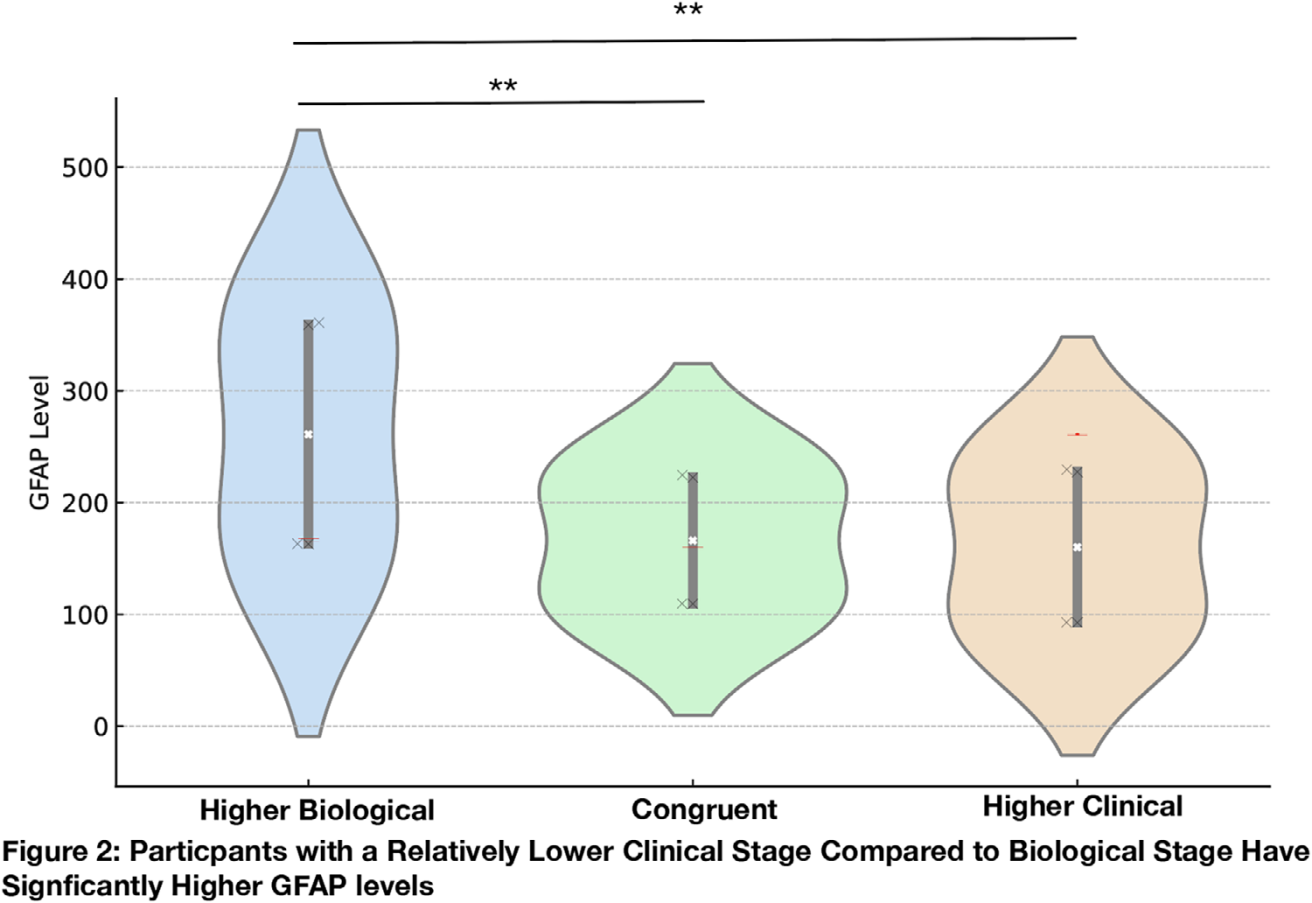# Evaluating Congruence Between Clinical and Biological Staging Across The Alzheimer’s Disease Spectrum

**DOI:** 10.1002/alz.093241

**Published:** 2025-01-03

**Authors:** Hussein Zalzale, Marina Scop Madeiros, Carolina Soares, Guilherme Bauer‐Negrini, Guilherme Povala, Pamela C.L. Ferreira, Livia Amaral, Firoza Z Lussier, Bruna Bellaver, Sarah Abbas, Matheus Scarpatto Rodrigues, Dana Tudorascu, Markley Oliveira, Cynthia Felix, Douglas Teixeira Leffa, Cécile Tissot, Cristiano Schaffer Aguzzoli, Emma Patrice Ruppert, Pampa Saha, Nesrine Rahmouni, Henrik Zetterberg, Kaj Blennow, Nicholas J. Ashton, Belen Pascual, Brian A. Gordon, William J. Jagust, Val J. Lowe, Thomas K Karikari, Hwamee Oh, William E Klunk, David N. Soleimani‐Meigooni, Pedro Rosa‐Neto, Suzanne L. Baker, Tharick A. Pascoal

**Affiliations:** ^1^ University of Pittsburgh, Pittsburgh, PA USA; ^2^ Lawrence Berkeley National Laboratory, Berkeley, CA USA; ^3^ Brain Institute of Rio Grande do Sul, PUCRS, Porto Alegre, RS Brazil; ^4^ McGill University, Montréal, QC Canada; ^5^ University of Gothenburg, Mölndal, Gothenburg Sweden; ^6^ Department of Psychiatry and Neurochemistry, Institute of Neuroscience and Physiology, The Sahlgrenska Academy, University of Gothenburg, Mölndal, Gothenburg Sweden; ^7^ Houston Methodist Research Institute, Houston, TX USA; ^8^ Washington University in St. Louis School of Medicine, St. Louis, MO USA; ^9^ University of California, Berkeley, Berkeley, CA USA; ^10^ Department of Radiology, Mayo Clinic, Rochester, MN USA; ^11^ Brown University, Providence, RI USA; ^12^ Memory and Aging Center, Weill Institute for Neurosciences, University of California, San Francisco, San Francisco, CA USA; ^13^ McGill University, Montreal, QC Canada

## Abstract

**Background:**

The newly proposed criteria by the AA working group incorporates both biological and clinical stages to characterize the progression of AD. In this study, we aim to evaluate the agreement between these two complementary systems.

**Methods:**

Using 188 participants from McGill TRIAD and 139 from the HEAD cohorts, we categorized participants into biological (0‐4) and clinical (0‐4) stages using amyloid PET, tau PET(MK‐6240), and clinical measures as described by the working group. Participants were then stratified into three categories: congruent (clinical = biological), higher in the biological stage (clinical < biological), and higher in the clinical stage (clinical > biological). In the TRIAD cohort, we further compared these groups for age, sex, years of education, vascular burden (WMH), microglial activation (PBR scan), Astrocyte reactivity (GFAP), amyloid load, tau load, and NPI‐Q scores using Welch two‐sided t‐test with FDR multiple comparison correction.

**Results:**

In the TRIAD cohort, 34% of participants were congruent, 24.5% had a higher clinical stage, and 41.5% had a higher biological stage. Meanwhile in the HEAD cohort 68.2% were congruent, 20.7% had a higher clinical stage, and 17% had a higher biological stage. TRIAD participants with a higher clinical stage had lower education (P = 0.02), more neuropsychiatric symptoms (P = 0.03), and a higher vascular burden (P = 0.03) (**Table 1**). As expected, people with higher biological stage had more amyloid(P<0.001), tau(P<0.001), and astrocyte reactivity(P<0.001) (**Table 1, Figure 2**).

**Conclusions:**

Our findings highlight important discordance between clinical and biological stages, which could be partially explained by cognitive reserve. This was supported by the protective effects of educational attainment in participants with a higher biological stage. Vascular burden played a major role in the cognitive impairment of individuals with higher clinical stages. Future studies should replicate these findings in larger more representative population‐based cohorts.